# Analysis of Attempted Suicide in a Pediatric Setting: Extracted Notes for Clinical Practice and Complexity of Help

**DOI:** 10.3390/ijerph19148604

**Published:** 2022-07-14

**Authors:** Sigita Lesinskienė, Odeta Kinčinienė, Rokas Šambaras, Emilijus Žilinskas

**Affiliations:** 1 Clinic of Psychiatry, Institute of Clinical Medicine, Faculty of Medicine, Vilnius University, 01513 Vilnius, Lithuania; rokas.sambaras@mf.vu.lt; 2 Clinic of Children’s Diseases, Institute of Clinical Medicine, Faculty of Medicine, Vilnius University, 01513 Vilnius, Lithuania; 3 Faculty of Medicine, Vilnius University, 01513 Vilnius, Lithuania; emilijus.zilinskas@mf.stud.vu.lt

**Keywords:** adolescents, attempted suicide, follow-up care

## Abstract

Background and Objectives: Suicidality among adolescents is a global mental health issue. However, the post-suicidal care of minors is insufficient and lacks complexity, as repeated attempts occur. Further, little is known about the social (i.e., family setting) and psychological (for example, exposure to bullying, suicidality, or addiction within a close environment) backgrounds of minors who engage in suicidal behavior in Lithuania. Thus, the aim of our study was to evaluate and compare psychosocial factors that may influence minors’ decisions to attempt suicide for the first time and then repeatedly and to extract notes for future clinical practice. Materials and Methods: Data from 187 cases of suicide attempts by minors treated at the Children’s Hospital of Vilnius University Santara Clinics from January 2011 to January 2018 was obtained and analyzed. *Results*: The data showed that 48.7% of minors hospitalized because of a suicide attempt had a history of previous suicide attempts. Minors who attempted suicide repeatedly were more often engaged in non-suicidal self-injurious behavior (*p* = 0.033). Further, a small number had experienced suicide within their close surroundings (*p* = 0.045). The comparative analysis did not reveal any significant differences in psychosocial backgrounds between first-time attempters and those who attempted suicide for at least a second time. Conclusions: Social and psychological support after a suicide attempt in a pediatric setting is lacking efficiency, as minors who repeatedly attempt suicide suffer from similar psychosocial burdens. Complex and targeted follow-up care is needed.

## 1. Introduction

Suicidal ideation and behavior among children and adolescents are significant global health issues. According to the data from the National Comorbidity Survey Replication Adolescent Supplement, the lifetime prevalence of adolescents’ suicidal ideation, plans, and attempts were 12.1%, 4.0%, and 4.1%, respectively [1]. In the US, 6.9% of 9th- to 12th-grade students reported at least one suicide attempt during the previous year [2]. Based on data from a study carried out in Lithuania, suicide makes up a quarter of all deaths in the population of adolescents aged 10–19 years [3].

Studies indicate that various psychological and social disadvantages minors face are significant predictors for suicide attempts: weak parental support, disturbed mother–child relationship, and lack of familial warmth [4,5]; improper family rearing behavior, separation from parents, and social problems among family members [6]; living in single-parent or reconstructed families [7,8]; living in care [9]; parental addiction to alcohol or other substances [10,11]; any participation in bullying (being a victim or perpetrator; cyber or physical bullying) [12,13,14,15]; non-suicidal self-injurious behavior [16]; or exposure to the suicide of a friend or family member [17,18]. However, the prevalence of such disadvantages varies among different countries and regions. For example, a study conducted in Lithuania emphasized negative family-related factors (i.e., single parenthood, foster care, or unfavorable family climate) to be especially burdensome for the mental health of children and adolescents [19]. Thus, more precise data regarding the psychosocial background of suicidal inpatient minors in Lithuania may be valuable for future comparative analyses conducted through other research.

Further, less is known about the psychosocial background of minors who repeatedly attempt suicide, as research mainly focuses on detecting risk factors for suicide attempts in general. Repeated suicide attempts by a child or adolescent partly reflects a lack of efficient and complex short- and long-term suicide prevention strategies. Thus, examining the psychosocial contexts of those who repeatedly attempt suicide may potentially highlight gaps in the follow-up care of suicide survivors.

Based on the abovementioned demand for data, we aimed to observe the psychosocial variables (i.e., family structure, presence or absence of parents’ addiction, exposure to bullying, non-suicidal self-injurious behavior patterns, and exposure to suicide in close surroundings) that are characteristic of minors who attempted suicide. Further, we sought to compare the prevalence of these variables among first-time attempters and those who attempted suicide repeatedly. Last, we aimed to extract notes that may be significant for organizing and implementing interdisciplinary follow-up care for suicide attempters in a pediatric setting.

## 2. Materials and Methods

Data for the cross-sectional retrospective study were obtained from the Children’s Hospital of Vilnius University Santara Clinics from January 2011 to January 2018. The inclusion criteria for the study were the patient’s age (under 18) and admission to a hospital after a suicide attempt (i.e., a potentially self-injurious behavior associated with at least some intent to die). The study included cases that met the ICD-10 criteria for Intentional self-harm (X70-84) and R45.81 Suicidal ideation. The ICD-10 codes for Intentional self-poisoning (X60-69) were not included, as poisoned patients are treated in another regional hospital in Vilnius. The exclusion criteria were a medical history of unintentional, accidental injuries (according to the ICD-10 Accidents (V01-X59) and Assault (X85-Y09)); deceased patient; lack of indication for hospitalization; and incomplete medical records. 

Data that were extracted from medical histories consisted of demographic variables (patient’s age and gender), variables concerning patient’s family structure (both parents vs. one parent at the moment of suicide attempt), patient’s living conditions—at home vs. foster care/orphanage, and other variables (experience of being bullied, parental alcoholism or other addiction, or suicide within a close environment). Data regarding the season during which the attempt occurred were also collected from medical histories. Signs of self-harm (any type of self-injurious behavior, including suicide attempts and non-suicidal self-injury) were collected and evaluated for medical examination protocols (cuts, burn signs on body, or scars left after self-harm). Circumstances of one’s self-harm, whether performed with or without suicidal intent, were evaluated by a psychologist or by a child and adolescent psychiatrist during the psychosocial assessment procedure. Self-harm without suicidal intent was identified as a non-suicidal self-injury. Data concerning the circumstances of the attempt (i.e., first attempt vs. relapse) were collected and evaluated using the “psychosocial assessment protocol for persons surviving a suicide crisis”. The protocol consists of general questions about a person’s age and gender. Further, the protocol consists of open questions about current suicidal thoughts, intentions, and previous suicidal experiences and was conducted by a psychologist or by a child and adolescent psychiatrist. The data from the medical histories and the “psychosocial assessment protocol for persons surviving a suicide crisis” were coded by selecting sentences, words, and phrases that corresponded to the psychosocial variables discussed in the aim of our study. For example, if the patient or their relatives indicated in the medical history that the child had experienced bullying, the case was marked as having experienced bullying; if the patient or their relatives indicated in the medical history that one of the parents had an addiction (e.g., alcohol and narcotic substances), the case was marked as having parental addiction; if the patient or their relatives noted in the medical history that the patient had faced suicide within a close environment, the case was marked as having suicide within a close environment. 

Continuous variables were expressed as the mean ± standard deviation, and qualitative data were reported as numbers and percentages. The normality of the variable distribution was tested by the Kolmogorov–Smirnov test. The significance of the differences between groups with a normal distribution of parameters was assessed by the Independent Samples *t*-test (compared children’s mean age by gender groups). Associations between qualitative parameters were tested using the χ^2^ test or Fisher’s exact test. The difference between the observed seasonal suicide attempt distribution and expected frequencies was tested using the χ^2^ test. The level of statistical significance was *p* < 0.05. Microsoft Excel 2010 was used for coding the procedures, and IBM SPSS 20.0 was used for statistical data analysis.

## 3. Results

A total of 187 cases were analyzed in the study. There were 160 (85.6%) cases involving girls and 27 (14.4%) involving boys. The mean age of minors was 15.04 (SD = 1.82), with 15.07 (SD = 1.75) for girls and 14.89 (SD = 2.19) for boys, (*p* = 0.405). The youngest girl at the moment of attempt was seven, and the youngest boy was eleven years old.

Most attempts were observed during spring (32.6%), followed by autumn (26.2%), winter (24.6%), and summer (16.6%) (χ^2^ = 5.095, *p* = 0.164). Almost half of minors (49.7%) admitted to having experienced bullying, 25.7% of minors had parents with an addiction to alcohol, and 16.6% had experienced suicide within their close surroundings. Only 40.1% of minors lived with both parents and 42.8% lived with one parent, followed by those living in orphanages (11.2%) and in foster care (5.9%). Finally, 74.3% of minors who attempted suicide were engaged in non-suicidal self-injurious behavior. 

**Figure 1:**
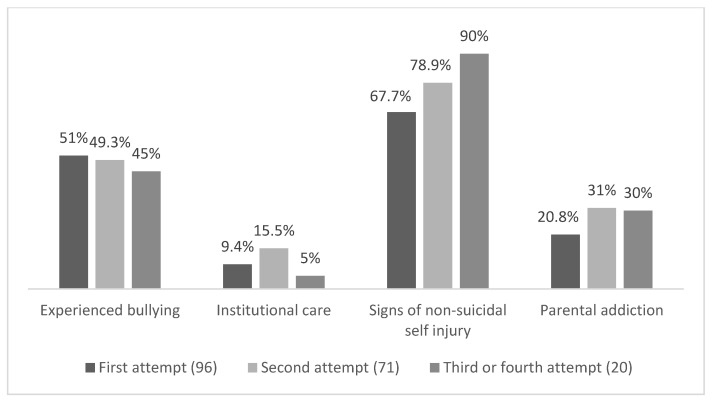
Descriptive statistics based on number of suicide attempts.

Ninety-six minors (51.3%) had attempted suicide for the first time and the mean age was 15.03 (SD = 1.78), while 91 (48.7%) had a history of previous suicide attempts and the mean age was 14.96 (SD = 1.92), *p* = 0.742. In the latter group, 74 (81.3%) minors had signs of non-suicidal self-injury and this was a significant difference compared to first-time attempters (67.7%, χ^2^(1) = 4.536, *p* = 0.033). Minors who had a history of a previous suicide attempt had experienced suicide within their close surroundings significantly less often (χ^2^(1) = 4.003, *p* = 0.045). The comparative analysis did not reveal any other significant differences between first-time attempters and those who attempted suicide for at least a second time. The main findings reflecting the differences are shown in Table 1 and Figure 1. 

Girls were found to be significantly more susceptible to non-suicidal self-injurious behavior than boys: 125 (78.1%) vs. 14 (51.9%), (χ^2^(1) = 7.520, *p* = 0.007). Seventy-four girls (46.3%) had attempted suicide for the second, third, or fourth time, while 17 (63.0%) boys had a history of previous suicide attempts (χ^2^(1) = 2.603, *p* = 0.107). 

The binary regression analysis indicated that a history of non-suicidal self-injury and female gender are predictive variables for repeated suicide attempts (beta = 1.095 and 1.042, *p* < 0.05, respectively); however, the potency of the regression model was insufficient (pseudo R^2^ = 0.082). 

## 4. Discussion

According to the literature review as well as our study results, suicidality in the pediatric population is a very real and sensitive problem [20]. Our study provided additional information regarding the social and psychological backgrounds of minors who had attempted suicide in Lithuania. Studies that assess the suicides of children and adolescents indicate that males more often die by suicide [21]. On the other hand, it is estimated that female adolescents tend to attempt suicide more often [22]. The sample of our study consisted of 85.6% female minors, which indicates similar trends in gender differences when attempting suicide. Further, based on our demographic data, the average age of children who attempted suicide was 15.04 (SD = 1.82). These data are in accordance with data from many other research studies in Europe and the US [1,23,24] but differ from data collected by African researchers, where the average age of a suicide attempt was found to be 17.5 years [25]. The peak period for hospitalizations due to suicide attempts was in spring. This finding is consistent with data extracted from a systematic review that indicated spring to be the season of most suicide attempts and not only of completed suicides [26]. The current tendency of seasonality is not yet well understood, and the reasons for this range from changes in availability to suicide methods [27], psychiatric disorders with a seasonal profile [28], and living in more rural areas [26] to biological mechanisms, such as variations in concentrations of sunshine-regulated hormones such as serotonin, melatonin, and vitamin D [29,30].

According to a systematic review and meta-analysis, children and young people who need to be provisioned for by the child welfare system are at an elevated risk of suicidal behavior [9]. Our results support such a measurement, as 11.2% of minors who attempted suicide lived in orphanages, even though the approximate number of people under 18 years of age and living in orphanages in Lithuania is only 0.8%. The latter finding highlights the need to implement significant changes in the social structure for raising children in Lithuania. Further, we found that only two fifths of minors lived with both parents. Similar results were found in another study conducted in France that examined adolescents who had attempted suicide [31]. As expected, an intact family structure may serve as a protective factor for a minor’s suicidality. Further, almost half of minors examined admitted that they had experienced bullying. The latter stressor seems to belong to the most significant predictors of suicide among adolescents [32]. This finding indicates an urgent need to implement anti-bullying programs, as they may be effective [33], at least in the short term [34]. A study from the U.S. highlighted that exposure to the suicidal behavior of a friend or family member is an equally significant risk factor for an adolescent’s suicidality or for being severely depressed [17]. Due to the insufficient potency of our regression model, we were not able to confirm such measurements. However, we believe that the number of minors in our study who experienced suicide within a close surrounding (16.6%) would be higher compared to community samples. Unexpectedly, minors who had friends or relatives that died by suicide engaged significantly less often in a repeated suicide attempt. Due to the limitations of our study design, the exact mechanism for the latter connection remains unclear.

Self-harm is common among minors in Lithuania as well as in other countries. A study performed in Lithuania in 2009 showed that more than 7.3% of Lithuanians aged under 20 have tried cutting themselves or have performed other forms of self-harm [35]. Studies that have examined non-suicidal self-injurious (NSSI) behavior within a community of high school samples reported a prevalence of between 15 and 25% [36,37,38]. Our study revealed that 67.7% of minors who were admitted to the intensive care unit due to a suicide attempt for the first time had signs of non-suicidal self-injury. Concomitant with other studies, this finding highlights that non-suicidal self-injurious behavior may be predictive for suicidal ideation and behavior [37]. Further, literature indicates that females are more likely to harm themselves than males [39]. Our study found similar data: non-suicidal self-harm for girls was statistically significantly more prevalent than for boys. Scientific articles show how patterns of self-harm vary by gender. Girls are more likely to cut themselves than boys, and they perceive self-harm as a strategy for coping with stress. Meanwhile, boys are more likely to harm themselves by deliberately consuming alcohol and taking drugs and are more likely to have problems with risky behaviors [40]. 

Despite the potential relationship between NSSI and suicidal behavior, we suggest observing NSSI in a wider perspective. For example, non-suicidal self-injurious behavior may be related to other mental health issues, such as negative body attitudes and disordered eating [41,42]. In addition, one study indicated NSSI to be associated with one’s hostility as well as verbal and indirect aggression [43]. As the detection of NSSI in a non-clinical community is hard to achieve, psychiatrists, psychologists, pediatricians, and other specialists dealing with suicidal adolescent inpatients should be aware of such comorbidities [44].

One of the aims of our study was to compare the psychosocial background of minors who attempted suicide for the first time and those who already had a history of suicide attempts. We found that repetitiveness is associated with non-suicidal self-injurious behavior. This measurement raises concerns that those minors who engage in NSSI behavior tend to be more determined to die by suicide even after the first attempt. On the other hand, experience with bullying, parental addiction, and living conditions were similarly prevalent in both minors who attempted suicide for the first time and in repeaters. Thus, the negative psychosocial contexts adolescents live in seem to be burdensome regardless of the number of suicide attempts. 

The impact of most psychosocial factors on suicide attempts in minors was summarized in our article. However, the complexity and specificity of help for prevalent mental health problems children and adolescents face is quite different in each country or culture [45,46]. Therefore, research is still very much needed. One of the key objectives is to share research and good practice interculturally. After reviewing the scientific literature, as well as analyzing the data from our study, we attempted to find and present key clinical notes that would be important to successfully organize and implement interdisciplinary help for suicidal minors.

Firstly, our study highlights a high number of minors who attempt suicide repeatedly. This finding may indicate gaps in the follow-up care of suicidal youths in Lithuania. According to data from the meta-analysis, the active follow-up of patients after a suicide-related crisis should be routine [47]. Further, it is indicated that brief and acute care and suicide prevention interventions reduce subsequent suicide attempts and increase linkage to follow-up care [48]. Thus, it is reasonable to implement active and acute care measures after a suicidal event to prevent a minors’ repeated suicidal attempts. Especially important is that as many people as possible who can understand and identify the potential signs or risks of a recurrent suicide attempt should be involved in the follow-up monitoring of adolescents. 

Further, most articles on suicide prevention state that gatekeeper programs are one of the most effective multipronged suicide prevention strategies [49]. The gatekeepers are everyday people equipped with the knowledge and skills to respond to someone at risk of suicide. It is especially important to develop gatekeeper programs that include a variety of professionals who work with young people. Particular attention should be focused on the development of gatekeeper programs in schools and day care centers because adolescents spend most of their time in school. Based on this, these types of programs are considered one of the most effective methods for addressing the problem of minors attempting suicide and for promoting help-seeking among youngsters [50]. However, it is also extremely important to increase parents’ knowledge of adolescent mental health, especially regarding the possible signs of suicidal behavior [51]. In addition, we observed that some of the minors who attempted suicide lived in foster care. Therefore, special attention should be focused on professionals who face the most vulnerable groups of minors (e.g., foster carers, social workers, and foster home staff).

Lastly, we emphasize that the stigma attached to mental health illnesses is a disturbing factor for those seeking mental health services. For example, Moskos et al. observed that adolescents who died by suicide were reluctant to admit to having mental health problems and were too embarrassed to seek help [52]. Thus, stigma-reducing measures with a focus on children and adolescents who possess burdensome psychosocial characteristics examined in our study are of great importance.

Our study has a few limitations. First, due to insufficient data from medical records, we were not able to include past medical history examination results into the evaluation process. For example, an adolescent who has a mood disorder or a substance-abuse problem may be more inclined towards suicidal behavior. Thus, the psychosocial variables examined in our study may be of second importance [53,54,55]. Further, we are aware that data regarding one’s acts of non-suicidal self-injuries is limited, as neither severity nor frequency of self-harm were examined. The latter aspects may have contributed to a more precise evaluation of the relationship between non-suicidal self-harm and suicidal behavior in the current inpatient population.

## 5. Conclusions

Our study provided additional information regarding the psychosocial profile of minors who are engaged in suicidal behavior in Lithuania. Family discord, institutional care, experiences of bullying, and suicides within a close environment are prevalent features in the primary care inpatient groups of minors after a suicide attempt. Signs of non-suicidal self-injury are significantly predictive of one’s repeated suicide attempts and require additional attention by physicians in a pediatric setting. Active follow-up care may serve as a protective measure for repeated suicide attempts by youths. 

## Figures and Tables

**Table 1:** Descriptive analysis of minors who attempted suicide for the first time and who already had history of suicide attempt(s).

	First Suicide Attempt (96 (51.3%))	Previous History of Suicide Attempt * (91 (48.7%))	*p* Value
Experienced bullying	51.0	48.4	0.713
Institutional care	9.4	13.2	0.409
Signs of non-suicidal self-injury	67.7	81.3	0.033
Parental addiction	20.8	30.8	0.120
Suicide in close surroundings	21.9	11.0	0.045

* Second, third, or fourth suicide attempt.

## Data Availability

Not applicable.
